# Editorial: Diagnosis and Treatment of Primary Aldosteronism: from Clinical Origin to Translational Research

**DOI:** 10.3389/fendo.2021.781105

**Published:** 2021-11-30

**Authors:** Wan-Chen Wu, Qiang Wei, Vin-Cent Wu

**Affiliations:** ^1^ Division of Endocrinology and Metabolism, Department of Internal Medicine, National Taiwan University Hospital, Taipei, Taiwan; ^2^ Department of Urology, Institute of Urology, West China Hospital, Sichuan University, Chengdu, China; ^3^ Department of Internal Medicine, National Taiwan University Hospital, Taipei City, Taiwan

**Keywords:** primary aldosteronism, adrenal venous sampling, MRA, APCCs, CYP11B2

Primary aldosteronism (PA) is one of the common causes of secondary hypertension, increasing the risk of cardiovascular disease and renal events as compared to essential hypertension, independently of blood pressure control (1–3).

Primary aldosteronism (PA) is one of the common causes of secondary hypertension, and is associated with higher risks of cardiovascular, renal, and metabolic sequelae, including left ventricular hypertrophy, myocardial infarction, atrial fibrillation, stroke, microalbuminuria, osteoporosis, as well as metabolic syndrome (1, 4–7).

The present Research Topic highlights the interplay between clinical diagnosis, underlying genetic etiologies, and clinical outcome PA. Overall, it focuses on the histopathologic findings, gene mutation, the coexistence of cortisol, cosecretion, and targeted treatments of PA.

Nanba et al. provide a template for researchers to study aldosterone-producing adrenal using formalin-fixed paraffin-material embedded sections for DNA capture, sequencing, and mutation determination.

Although cortisol cosecretion in aldosterone-producing adenoma (APA) has been reported (8), the clinical relevance of such APA coexisting with cortisol-producing adenoma has not been illustrated (8). Inoue et al. summarize the current state of knowledge about cortisol cosecretion with PA. They conclude, there is increasing evidence about the relatively high prevalence of cortisol cosecretion in PA and its potential influence on adverse health outcomes.

Adrenal venous sampling (AVS) is the test of choice to identify patients with a surgically curable subtype of PA (9). Okamoto et al. assess 1586 PA patients without apparent adrenal tumors in the multicenter study and conclude adrenal venous sampling should be considered for male hypokalemic PA patients with high ARRs because of the rates of the lateralized subtype and cardiovascular events are high in these patients.

The major advance of understanding PA pathophysiology is the identification of several somatic driver mutations in ion channels and ATPases in lateralized aldosterone-producing adenoma (APA) (10). Utilizing the immunohistochemical (IHC) detection of aldosterone synthase (CYP11B2) has allowed the identification of aldosterone-producing cell clusters (APCCs) with unique focal localization positive for CYP11B2 expression in the subcapsular portion of the human adult adrenal cortex. Pauzi and Azizan reviewed APCCs associated aldosterone-stimulating somatic gene mutations (recently replaced by aldosterone-producing micronodules) and their accumulation during the aging process, raising the possibility that APCCs may play a role in the development of PA and age-related hypertension.

Recent studies indicate that somatic mutations of the potassium channel *KCNJ5* gene could be identified in 34 to 73% of APAs (11). Wang H. et al. find that gender, duration of hypertension, and the highest systolic blood pressure were independent predictors for the postoperative cure of APA identified based on HISTALDO histopathologic groups.

Lu et al. demonstrated that NP-59 adrenal scintigraphy could predict *KCNJ5* mutations in PA patients by two semiqualitative parameters [adrenal to liver ratio (ALR) and lesion to the contralateral ratio of bilateral adrenal glands (CON)] and provided more information in an individualized treatment plan.

In regarding possible variations in response to hormonal stimuli, APAs with ATPase-mutations are more responsive to ACTH than KCNJ5-mutated APAs was found in Lim et al. study.

PA patients also have a higher risk of cardiovascular diseases and greater cardiac remodeling compared to those with essential hypertension (7, 12). Pan et al. demonstrate extensive cardiac remodeling in APA patients through hemodynamic and non-hemodynamic causes. Adrenalectomy improved both hemodynamic and non-hemodynamic components of left ventricular remodeling, which also correlated with decreases in blood pressure and ARR.

Zhou et al. report the severity of diastolic dysfunction independently relates to the degree of diffuse myocardial fibrosis in PA patients with elevated aldosterone level.

The excess aldosterone causes atrial structural and electrical remodeling, which induce atrial fibrillation genesis, and PA was associated with a higher incidence of new-onset atrial fibrillation (NOAF) that could ameliorate after adrenalectomy (13). Tsai et al. performed a meta-analysis and showed different effects of PA treatment on NOAF risk. The PA patients receiving MRA treatment had a higher risk of NOAF compared to the PA patients receiving adrenalectomy and the patients with essential hypertension.

Chen et al. provide new insights into the relationship between adipose tissue and aldosterone excess in patients with APA and idiopathic hyperaldosteronism (IHA). Abdominal adiposity indexes were similar in patients with IHA and those with essential hypertension but were markedly lower in patients with APA. Aldosterone-to-renin ratio (ARR) was negatively correlated with abdominal adiposity indexes in patients with APA but not in patients with IHA.

The endocrine-gut interaction show PA patients had fewer short-chain fatty acids-producing genera and more inflammation-associated genera than healthy controls. Alteration of gut microbiota may contribute to the obesity and diabetics status in PA, as shown by Liu et al.

Some studies showed similar long-term cardiac effects of surgical or medical treatment in PA patients (14). However, other studies showed a lower incidence of adverse cardiovascular outcomes in PA patients treated by adrenalectomy (15). Huang et al. show superior performance of surgical treatment over medical treatment for patients with lateralized PA on both composite and individual clinical cardiovascular outcomes in this meta-analysis. An interesting study presented by Tezuka and Turcu on targeted treatment, they identified that lower baseline serum potassium, lower mineralocorticoid receptor antagonist (MRA) doses, and beta-blocker use was independently associated with lower odds of achieving target renin in PA. Their findings suggest that renin targets, when PRA was <1.0 ng/mL/h and DRC was <8.0 pg/mL, are followed in very few and are achieved in under half of such PA patients seen in an academic setting, with possibly even lower rates in community practices.

In the future, Wang C. et al. conclude international collaboration on the clinical and molecular mechanism of PA will be important to improve the investigation and therapy of patients with PA.

Highlighting the novel insights into the possible mechanism and outcome of PA, all the studies in this special issue fill some gaps of knowledge and give future challenges for research in this field.

## Author Contributions

W-CW wrote the draft of the article. V-CW and QW supervised the results. All authors contributed to the article and approved the submitted version.

## Funding

This study was supported by MOST 107-2314-B-002-026-MY3, 108-2314-B-002-058, 109-2314-B-002-174-MY3], National Taiwan University Hospital [109-S4634, PC-1264, PC-1309, VN109-09, UN109-041, UN110-030] Grant MOHW110-TDU-B-212-124005 and Mrs. Hsiu-Chin Lee Kidney Research Fund. The NGS experiments were performed by the NGS Core Laboratory, Molecular Medicine Research Center, Chang Gung University, Taiwan (grant CLRPD1J0012).

## Conflict of Interest

The authors declare that the research was conducted in the absence of any commercial or financial relationships that could be construed as a potential conflict of interest.

## Publisher’s Note

All claims expressed in this article are solely those of the authors and do not necessarily represent those of their affiliated organizations, or those of the publisher, the editors and the reviewers. Any product that may be evaluated in this article, or claim that may be made by its manufacturer, is not guaranteed or endorsed by the publisher.
